# Predictors of successful recanalization following endovascular intervention in non-acute basilar artery occlusion

**DOI:** 10.3389/fneur.2025.1496852

**Published:** 2025-04-07

**Authors:** Ying Liu, Zuoli Wu, Shengwei Wei, Wenbo He, Weihao Ye, Shang Xu, Baozi Huang, Chao Qin, Wen Gao, Ziming Ye

**Affiliations:** ^1^Department of Rehabilitation Medicine, The Second Affiliated Hospital of Guangxi Medical University, Nanning, China; ^2^Department of Neurology, The First Affiliated Hospital of Guangxi Medical University, Nanning, China; ^3^Jiangbing Hospital, Guangxi Zhuang Autonomous Region, Nanning, Guangxi, China; ^4^Department of Neurology, Liuzhou People's Hospital Affiliated to Guangxi Medical University, Liuzhou, Guangxi, China

**Keywords:** non-acute basilar artery occlusion, endovascular intervention, recanalization, predictors, occlusion length, collateral compensation

## Abstract

**Objective:**

This study aims to identify factors influencing successful recanalization following endovascular intervention for non-acute basilar artery occlusion (NABAO).

**Background:**

Endovascular treatment (EVT) is a feasible approach for treating non-acute basilar artery occlusion, but it presents significant technical challenges due to the lack of standardized treatment protocols. Therefore, identifying patients most likely to benefit is critical to minimizing procedural risks.

**Methods:**

A retrospective analysis was conducted on 115 patients with NABAO treated via EVT. Factors associated with successful recanalization, including clinical symptoms, demographic characteristics, procedural outcomes, and imaging findings, were analyzed using multivariate analysis. A scoring system was developed based on independent predictors.

**Results:**

Successful recanalization (defined as modified Thrombolysis in Cerebral Infarction [mTICI] ≥2b) was achieved in 81.7% (94/115) of cases. Multivariate analysis revealed that occlusion duration >3 months (odds ratio [OR]: 0.187, 95% confidence interval [CI]: 0.051–0.688, *p* = 0.012), blunt-shaped occlusion ends (OR: 0.236, 95% CI: 0.072–0.777, *p* = 0.018), occlusion length > 30 mm (OR: 0.144, 95% CI: 0.031–0.669, *p* = 0.013), and insufficient or absent distal compensation (OR: 0.25, 95% CI: 0.075–0.835, *p* = 0.024) were independent predictors of reduced technical success. The receiver operating characteristic (ROC) curve index for the scoring system, based on these independent predictors, was 0.817 (95% CI: 0.698–0.936, *p* < 0.001), with a sensitivity of 71.4% and a specificity of 85.4% at a cutoff of 2.5 points.

**Conclusion:**

Longer occlusion duration (>3 months), blunt-shaped occlusion ends, occlusion length > 30 mm, and insufficient distal collateral compensation are independent negative predictors for successful recanalization in patients with NABAO treated via EVT. The proposed scoring system can help screen patients suitable for treatment and optimize treatment strategies, but further validation in prospective cohorts is needed.

## 1 Introduction

Stroke remains a leading cause of disability and mortality worldwide, with acute ischemic stroke (AIS) accounting for nearly 80% of all cases (1–3). Early intervention to restore blood flow and salvage the ischemic penumbra is critical for improving outcomes in AIS (4, 5). The current gold-standard treatments for early recanalization include intravenous tissue plasminogen activator (rt-PA) thrombolysis and mechanical thrombectomy (6, 7). However, the narrow therapeutic time window limits the number of patients who are eligible for these interventions, leaving a substantial proportion untreated (8).

The efficacy of endovascular treatment (EVT) in acute posterior circulation large vessel occlusion has been well established. A recent meta-analysis revealed that for patients with acute vertebrobasilar artery occlusion presenting moderate to severe symptoms, EVT significantly increases the likelihood of achieving favorable functional outcomes by approximately 2.5-fold and markedly reduces overall disability and mortality rates (9). For adult patients with basilar artery occlusion (BAO)-related acute ischemic stroke presenting within 24 h from last known well, current guidelines recommend EVT combined with best medical therapy (BMT) over BMT alone, with this recommendation applies only to patients with a National Institutes of Health Stroke Scale (NIHSS) score of 10 or higher (10). However, for patients with BAO who fall outside the recommended time window for intervention, no consensus exists regarding optimal treatment strategies. Current international guidelines continue to recommend antiplatelet therapy, statins, lifestyle modification, and aggressive management of vascular risk factors, yet clinical outcomes remain suboptimal with these approaches (11, 12). Emerging evidence from single-center studies suggests that EVT for non-acute basilar artery occlusion (NABAO) may be feasible, offering potential benefits in restoring perfusion and improving clinical prognosis (13). Nonetheless, the procedural risks remain high, and success rates are inconsistent, primarily due to heterogeneous occlusion characteristics (e.g., chronicity, morphology) and the absence of validated selection criteria. Unlike Acute Basilar Artery Occlusion (ABAO), where thrombus composition and timing dominate decision-making, NABAO requires distinct predictors to balance procedural risks (e.g., vessel dissection, hemorrhage) against potential benefits.

Current literature on NABAO EVT is limited to small retrospective cohorts (14), with no consensus on which patients are optimal candidates. Key questions remain unanswered: How do occlusion duration, morphology, and collateral status interact to influence recanalization success? Can a standardized scoring system guide clinicians in selecting patients most likely to benefit from EVT?

A major challenge of NABAO EVT lies in identifying patients who are most likely to benefit from EVT and who have a high likelihood of successful recanalization. This study presents a retrospective analysis of NABAO treated with endovascular recanalization, aiming to identify predictors of successful outcomes and to develop a scoring system to aid in patient selection for intervention.

## 2 Methods

We conducted a retrospective, multi-center study of NABAO recanalization treatment from January 2019 to February 2024. Basilar artery occlusion was evaluated using computed tomography angiography (CTA) and confirmed by digital subtraction angiography (DSA). Non-acute basilar artery occlusion was defined as occlusion diagnosed more than 24 h after symptom onset (14). All patients underwent endovascular intervention following the signing of informed surgical consent.

### 2.1 Inclusion criteria

(1) Age > 18 years.
(2) Duration of basilar artery occlusion >24 h.
(3) Basilar artery occlusion confirmed by DSA.
(4) Consent for endovascular recanalization provided by the patient or their family members, following detailed explanation of the procedure and risks (14).

### 2.2 Exclusion criteria

(1) Patients diagnosed with NABAO via DSA but who declined endovascular intervention, either personally or via family members.
(2) Severe cardiac insufficiency or significantly impaired liver and kidney function, rendering the patient unfit for surgery.
(3) Patients with cachexia, untreated malignant tumors, or an expected life expectancy of less than 2 years.
(4) Patients with non-atherosclerotic causes of occlusion, including arterial dissection, cardiogenic embolism, vasculitis, history of radiotherapy, or congenital vascular malformations.
(5) History of cerebral hemorrhage.
(6) Patients with a high risk of bleeding or contraindications to antiplatelet therapy.
(7) Recent gastrointestinal bleeding, major surgical procedures, or fever within the past month that had not been effectively controlled.
(8) Patients with active sepsis where blood cultures remained positive (14).

### 2.3 Interventional techniques

#### 2.3.1 Preoperative preparation

All patients were placed on dual antiplatelet therapy (DAPT) for 5 days before the procedure. This regimen included aspirin (100 mg daily) and clopidogrel (75 mg daily) (15). Additionally, patients received 40 mg of atorvastatin daily to stabilize atherosclerotic plaques and lower lipid levels.

#### 2.3.2 Surgical procedure

All procedures were performed under general anesthesia by experienced neurointerventional surgeons. Each surgeon had at least 5 years of experience in neurointerventional procedures, including the treatment of chronic occlusions of large intracranial vessels (minimum of 5 cases) or emergency thrombectomy (at least 10 cases). The annual caseload for intracranial stent placements exceeded 10 cases per surgeon in the previous 3 years (16).

During the procedure, an initial dose of 3,000 units of heparin was administered intravenously, with 1,000 additional units given every hour to maintain anticoagulation. A 5F or 6F intermediate catheter was used to access the target vertebral artery. An intracranial microcatheter and neuroguidewire were employed to attempt passage through the occluded segment. If the guidewire failed to cross the occlusion after 30 min of attempts, if the cumulative contrast volume exceeded 300 mL, or if the guidewire penetrated the vessel wall, the procedure was considered a failure of recanalization (17).

If successful, the microcatheter was advanced through the occlusion, and intravascular angiography was performed to confirm that the guidewire was within the true lumen. Subsequently, a 300 cm exchange guidewire was placed, and a 1.5 or 2.0 mm intracranial balloon was used for pre-dilation of the occlusion. If there was no elastic recoil or vascular dissection at the stenosis site, stenting was not required. However, if recoil or dissection occurred, an appropriately sized balloon-expandable or self-expanding stent was deployed. If the stent did not fully expand, post-dilation was performed. A recanalization was deemed successful if the final blood flow reached a modified Thrombolysis in Cerebral Infarction (mTICI) grade of 2b or higher (18).

### 2.4 Postoperative management

Nimodipine was administered postoperatively to prevent vasospasm, and blood pressure was strictly maintained between 110–120/60–80 mmHg. DAPT was continued for 3 to 6 months after the procedure, followed by single antiplatelet therapy. Long-term lipid-lowering therapy with statins was prescribed to maintain stable cholesterol levels.

### 2.5 Imaging assessment

Surgical outcomes and lesion imaging characteristics were independently assessed by two neurointerventional specialists. In cases of disagreement, a third independent expert reviewed the images to reach a consensus. The presence of contrast filling at the distal end of the occlusion was defined as a stump, which was classified as either conical or blunt. Occlusion length was measured on lateral DSA images, from the point of occlusion to the distal reconstitution site, excluding curvature in the vessel. Occlusions were categorized as ≤30 mm or > 30 mm. If the distal end was not visible, the occlusion length was considered >30 mm. On lateral DSA, two lines were drawn from the confluence of the vertebral arteries to the basilar artery and from the established access point to the vertebral artery, with the angle formed between these lines recorded as the vertebro-basilar artery (VA-BA) non-planar angle (19).

### 2.6 Statistical analysis

Statistical analyses were conducted using IBM SPSS Statistics version 25.0 (SPSS Inc., Chicago, IL, USA). Categorical variables were presented as counts and percentages, and compared using the chi-square test, continuity-corrected chi-square test, or Fisher’s exact test. Continuous variables were tested for normality using the Kolmogorov–Smirnov test. Data following a normal distribution were expressed as mean ± standard deviation (SD) and compared using the independent t-test, while non-normally distributed data were reported as median and interquartile range (IQR) and compared using the Mann–Whitney U test. A *p*-value ≤0.05 was considered statistically significant.

Logistic regression was performed for variables with *p* ≤ 0.05 in univariate analysis, adjusted for age and gender. Multivariate logistic regression was then used to identify independent predictors, and scores were assigned based on the coefficients of significant variables. Receiver operating characteristic (ROC) curves were used to evaluate model performance, with the Youden index determining sensitivity and specificity. Area under the curve (AUC) values were interpreted as follows: AUC ≤0.5 (low), 0.7 < AUC ≤0.9 (moderate), and AUC >0.9 (high).

## 3 Results

A total of 115 patients with symptomatic NABAO underwent endovascular treatment for recanalization. Among the patients, 78 were male (67.2%) and 37 were female (32.2%), with an age range of 41 to 84 years (mean age 63.01 ± 9.47 years). The overall recanalization success rate was 81.7% (94/115). Demographic and lesion characteristics are summarized in Tables 1, 2.

**Table 1:** Demographic characteristics.

	Failure group (*N* = 21)	Success group (*N* = 94)	Total (*N* = 115)	*p* value
Age (years)	63.19 ± 7.731	62.97 ± 9.848	63.01 ± 9.466	0.923
Male (%)	18 (85.7)	60 (63.8)	78 (67.8)	0.052
Hypertension (%)	15 (71.4)	73 (77.7)	88 (75.5)	0.746
Diabetes mellitus (%)	6 (28.6)	34 (36.2)	40 (34.8)	0.44
Hyperlipidemia (%)	7 (33.3)	31 (33.0)	38 (33.0)	0.975
Hyperhomocysteinemia (%)	7 (33.3)	27 (28.7)	34 (29.6)	0.676
Smoking (%)	6 (28.6)	13 (13.7)	19 (16.5)	0.187
Coronary artery disease (%)	5 (23.8)	11 (11.7)	16 (13.9)	0.271
Total Cholesterol mg/dL	168.47 ± 41.70	182.06 ± 50.01	179.58 ± 4.72	0.249
Creatinine mg/dL	0.94 (0.80–1.050)	0.845 (0.67–1.01)	0.86 (0.70–1.03)	0.105
Estimated Glomerular Filtration Rate ml/min	84.87 ± 25.40	91.60 ± 30.39	90.36 ± 29.55	0.348

Estimated glomerular filtration rate, calculated from the diet change in renal disease (MDRD) formula.

**Table 2:** Lesion characteristics.

	Failure group (*N* = 21)	Success group (*N* = 94)	Total (*N* = 115)	*p* value
Calcification over occluded segment (%)	3 (14.3)	9 (9.60)	12 (10.4)	0.807
Occlusion stump condition				
Tapered (%)	7 (33.3)	70 (74.5)	77 (67.00)	< 0.001
Blunt (%)	14 (66.7)	24 (25.5)	38 (33.0)	
Angle of basilar artery and vertebral artery >45° (%)	10 (47.6)	30 (31.9)	40 (34.8)	0.172
Occlusion length > 30 mm (%)	6 (28.6)	6 (6.4)	12 (10.4)	0.009
Occlusion site				
BA distal (%)	4 (19.05)	12 (12.77)	16 (13.91)	0.280
BA mid-end (%)	8 (38.10)	22 (23.40)	30 (26.09)	
BA proximal (%)	9 (42.85)	60 (63.83)	69 (60.00)	
Good collateral circulation (%)	9 (42.9)	62 (66.0)	71 (61.7)	0.049
Time from diagnosis of vessel occlusion to endovascular intervention				
≤ 3 months	9 (42.9)	75 (79.8)	84 (73.0)	0.001
> 3 months	12 (57.1)	19 (20.2)	31 (27.0)	

### 3.1 Univariate analysis

Univariate logistic regression analysis was performed to identify factors associated with successful recanalization. The odds ratio (OR) and 95% confidence interval (CI) for blunt-type vessel occlusion were 0.17 (95% CI: 0.060–0.481, *p* = 0.001), for occlusion length > 30 mm were 0.15 (95% CI: 0.040–0.566, *p* = 0.005), for absence or insufficiency of collateral circulation were 0.345 (95% CI: 0.128–0.933, *p* = 0.036), and for time from diagnosis to surgery >3 months were 0.202 (95% CI: 0.07–0.586, *p* = 0.003). These findings suggest that each of these factors was associated with a lower likelihood of successful recanalization. The results of the univariate analysis are detailed in Table 3.

**Table 3:** Single factor logistic regression analysis of successful factors for intravascular recanalization.

	OR (95% Cl)	*p* value
Hypertension	0.658 (0.217–1.997)	0.46
Diabetes mellitus	0.816 (0.279–2.385)	0.71
Hyperlipidemia	1.067 (0.383–2.976)	0.901
Hyperhomocysteinemia	1.089 (0.388–3.057)	0.871
Smoking history	1.974 (0.599–6.498)	0.263
Coronary heart disease	1.281 (0.311–5.288)	0.732
Total cholesterol	1.007 (0.996–1.018)	0.207
Creatinine	1.056 (0.423–2.632)	0.908
Estimated Glomerular Filtration Rate	1.006 (0.989–1.024)	0.492
Calcification at the occlusion site	1.529 (0.333–7.012)	0.585
Blunt	0.17 (0.060–0.481)	**0.001**
Angle of basilar artery and vertebral artery >45°	1.699 (0.638–4.530)	0.289
Occlusion length > 30 mm	0.15 (0.040–0.566)	**0.005**
Occlusion site		
BA distal	1.173 (0.219–6.288)	0.852
BA mid-end	1.443 (0.364–5.716)	0.601
BA proximal	0.506 (0.166–1.548)	0.233
Absent or insufficient distal collateral compensation	0.345 (0.128–0.933)	**0.036**
Time from diagnosis of vascular occlusion to surgery >3 months	0.202 (0.07–0.586)	**0.003**

OR, Odds Ratio; CI, Confidence Interval. Bold values indicate statistically significant results (*p* < 0.05).

### 3.2 Multivariate analysis

Multivariate logistic regression analysis, adjusted for age and gender, was conducted on variables with *p* ≤ 0.05 in univariate analysis. These variables included blunt morphology of the occlusion, occlusion length > 30 mm, absence or insufficiency of distal collateral compensation, and time from diagnosis to surgery >3 months. The analysis identified the following as negative predictors of successful recanalization: blunt occlusion morphology (OR: 0.236, 95% CI: 0.072–0.777), occlusion time > 3 months (OR: 0.187, 95% CI: 0.051–0.688), occlusion length > 30 mm (OR: 0.144, 95% CI: 0.031–0.669), and absence or insufficiency of distal collateral compensation (OR: 0.25, 95% CI: 0.075–0.835). These results are shown in Table 4.

**Table 4:** Multivariate logistic regression analysis after adjusting for gender and age.

	Coefficient	OR (95% CI)	*p* value
Gender	−0.868	0.42 (0.091–1.938)	0.266
Age	0.015	1.015 (0.948–1.086)	0.678
Operation time > 3 months	−1.678	0.187 (0.051–0.688)	**0.012**
Blunt	−1.443	0.236 (0.072–0.777)	**0.018**
Occlusion length > 30 mm	−1.936	0.144 (0.031–0.669)	**0.013**
Absent or insufficient distal collateral compensation	−1.385	0.25 (0.075–0.835)	**0.024**

OR, Odds Ratio; CI, Confidence Interval. Bold values indicate statistically significant results (*p* < 0.05).

### 3.3 Basilar artery occlusion scoring system

**Figure 1:**
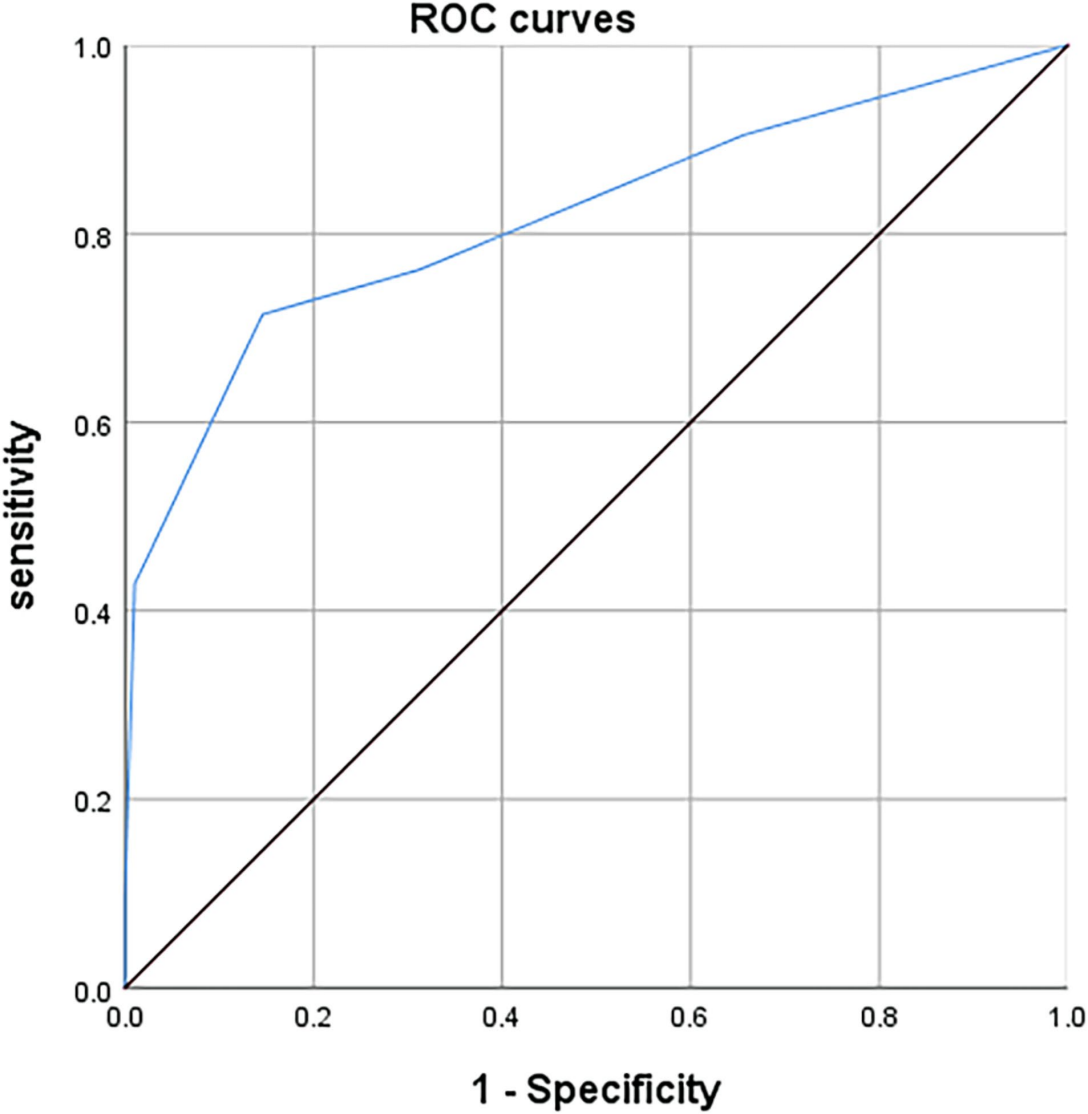
ROC curve of the scoring scale for successful recanalization by endovascular interventional treatment. The c-index on the based of area under the curve for basilar artery occlusion scoring system in predicting successful recanalization by endovascular interventional treatment was 0.817 (95% CI: 0.698–0.936, *p* < 0.001).

A predictive scoring system was developed based on the coefficients from the multivariate analysis. The point assignment for each predictor was determined by rounding the absolute values of their multivariate logistic regression coefficients to the nearest integer. Time from diagnosis to surgery >3 months was assigned 2 points, blunt occlusion morphology was assigned 1 point, occlusion length > 30 mm was assigned 2 points, and absence or insufficiency of distal collateral compensation was assigned 1 point. The total score for each patient ranged from 0 to 6 points. A receiver operating characteristic (ROC) curve was generated using this scoring system (Figure 1). The Youden index was calculated to be 0.568, and the area under the curve (AUC) was 0.817 (95% CI: 0.698–0.936, *p* < 0.001). The optimal cutoff score was 2.5 points. Patients with a score of 0–2 had a higher likelihood of successful recanalization, with a sensitivity of 0.714 and specificity of 0.854.

### 3.4 Example case

**Figure 2:**
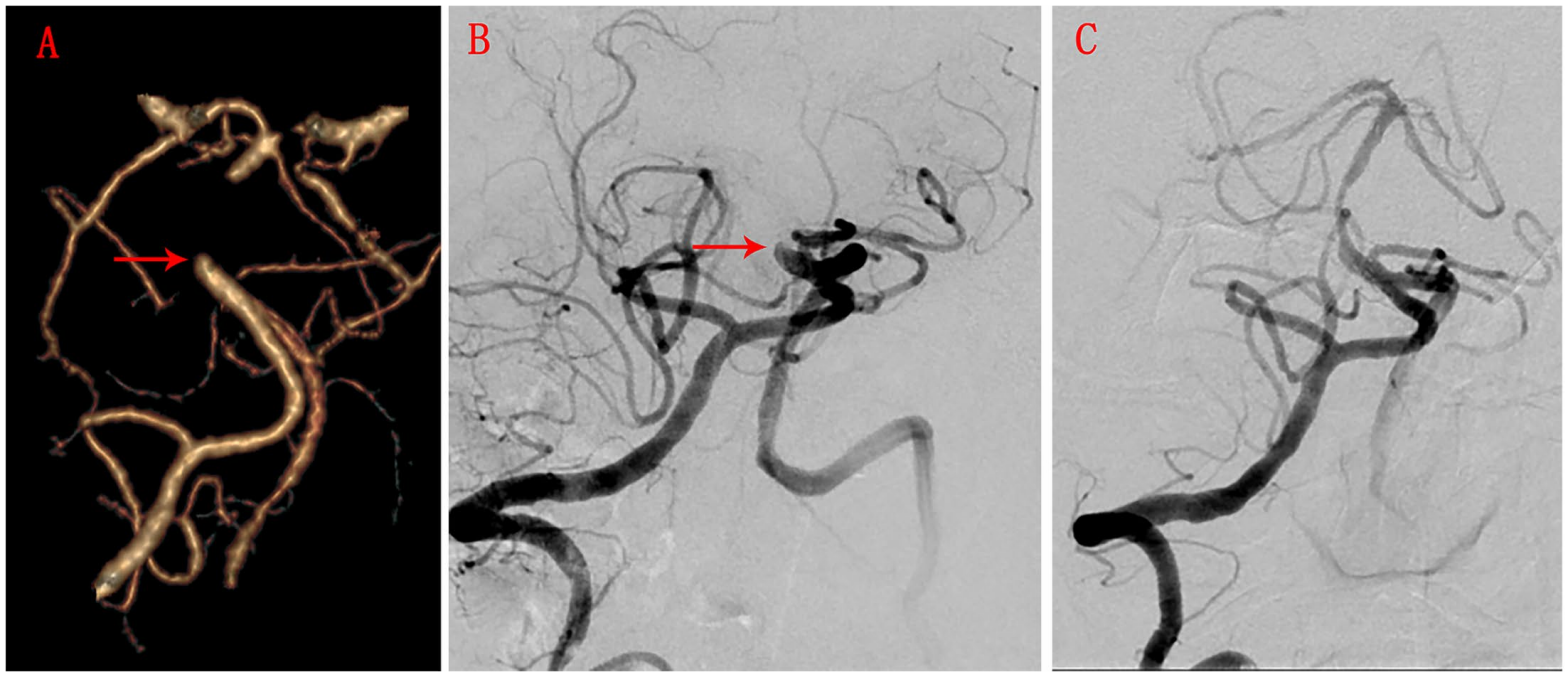
CTA and DSA images in case 1. **(A)** CTA shows the degree of basilar artery occlusion, the shape of the stump (red arrow), collateral circulation compensation, and the length of occlusion. **(B)** BSA shows the degree of basilar artery occlusion, the shape of the stump (red arrow), collateral circulation compensation, and the length of occlusion. **(C)** BSA after successful recanalization by endovascular interventional therapy.

An 84-year-old female presented with speech impairment for 4 days, which worsened with dysphagia over the last 2 days (0 points for symptoms). CTA and DSA indicated proximal and mid-basilar artery occlusion (Figures 2A,B), with a blunt stump morphology (1 point) (Figures 2A,B, red arrow), and good collateral compensation to the distal occlusion site (0 points). The occlusion length was <30 mm (0 points), resulting in a total lesion score of 1 point. Based on the scoring system, the patient had a high likelihood of successful endovascular recanalization (Figure 2C).

## 4 Discussion

Recent studies indicate that non-acute occlusions of large intracranial arteries are more prevalent among Asians, Hispanics, and African Americans (20). BAO occurs in approximately 1.2–2.5% of strokes, making it the most common site of posterior circulation vascular occlusion (21–23). Currently, there are no established domestic or international guidelines for the EVTof NABAO. Symptomatic intracranial artery occlusion carries an annual stroke risk of up to 23.4% (24, 25). A non-randomized controlled study on posterior circulation bypass surgery demonstrated positive outcomes; however, surgical revascularization of the posterior circulation is more challenging than that of the anterior circulation, necessitating experienced centers and surgeons. Additionally, the study had a limited sample size (with only 12 BAO patients enrolled) (26). Consequently, this procedure has not been widely adopted. The evolution of EVT for ABAO has been shaped by conflicting evidence from early and recent clinical trials. Early randomized controlled trials (RCTs), such as the Endovascular Treatment versus Standard Medical Treatment for Basilar Artery Occlusion (BEST) trial and the Basilar Artery International Cooperation Study (BASICS), did not demonstrate the superiority of EVT combined with BMT despite numerical trends favoring thrombectomy (22, 27). Indeed, the high crossover rate in the BEST trial and the lack of continuous enrollment in BASICS limited the certainty of their results. Recent RCTs, such as ATTENTION and BAOCHE, focused on patients with moderate-to-severe neurological deficits (NIHSS ≥10) and extended the treatment time window to 24 h. These trials clearly demonstrated the significant advantages of EVT: the ATTENTION trial (46% vs. 23%) and the BAOCHE trial (46% vs. 24%) found that the proportion of patients achieving a mRS score of 0–3 at 90 days was significantly higher in the EVT group compared to the BMT group for acute BAO patients within 12 h and 6–24 h from symptom onset, respectively (18, 28). Finally, a meta-analysis of the aforementioned four studies revealed that, for stroke patients treated within 12 h of onset, endovascular therapy significantly improved rates of favorable functional recovery (45% vs. 30%) and functional independence (35% vs. 21%) at 90 days, reduced mortality (36% vs. 45%) and overall disability severity, but increased the risk of symptomatic intracranial hemorrhage (5% vs. <1%) compared to the control group. Additionally, treatment benefits were more pronounced in patients with baseline NIHSS ≥10 and those with proximal vascular occlusions (9). These findings align with the updated European Stroke Organization (ESO) guidelines recommending EVT + BMT for BAO within 24 h in eligible patients, emphasizing the critical role of time-sensitive, imaging-guided decision-making (10). Its efficacy is comparable to that of EVT for anterior circulation large vessel occlusions, providing new evidence-based support for the treatment of basilar artery occlusion.

Li et al. retrospectively analyzed 18 patients with non-acute dominant vertebrobasilar artery occlusion and compared them with 32 patients with acute atherosclerotic vertebrobasilar artery occlusion undergoing EVT (29). Successful recanalization was achieved in 16 cases (88.9%) of non-acute occlusion and 30 cases (93.8%) of acute occlusion (*p* = 0.948), indicating no significant difference between the groups. Furthermore, the modified Rankin Scale (mRS) scores at 90 days post-procedure were comparable, suggesting that EVT for non-acute occlusion yields similar clinical outcomes and success rates as for acute occlusion.

However, recanalization of basilar artery occlusions remains technically challenging due to variations in occlusion length and access vessel curvature. Complications at the occlusion site, such as hemorrhage and vascular dissection, can lead to catastrophic outcomes. Therefore, comprehensive preoperative evaluation, particularly of lesion characteristics, is essential for predicting recanalization success and aiding operators in assessing the feasibility of occlusion reopening.

### 4.1 Occlusion time and technical success rate

Previous studies suggest that EVT within three months of occlusion onset may be feasible for patients with intracranial artery occlusion (30, 31). The success rate of vascular recanalization decreases as the duration of occlusion increases due to thrombus organization and fibrosis, leading to calcified plaque formation and increased difficulty in traversing the occlusion with microguidewires (32). Currently, there is no consensus on the optimal timing for EVT in NABAO, and further multicenter randomized studies are needed to provide high-quality evidence. Our findings also suggest that a longer duration of occlusion is associated with a lower likelihood of successful recanalization.

### 4.2 Vascular occlusion residual end morphology and length

A multicenter retrospective study on EVT for symptomatic non-acute extracranial vertebral artery occlusion indicated that the morphology of the occlusion stump is associated with recanalization difficulty (33). A tapered (conical) stump facilitates smoother guidewire passage through the occlusion, whereas a blunt stump decreases the success rate of guidewire traversal. Our study also highlighted that occlusions longer than 30 mm have lower success rates, possibly due to increased difficulty in guidewire navigation and a higher risk of vessel wall injury. These findings suggest that both longer lesion length and blunt occlusion morphology are negative predictors for successful EVT recanalization. Such information is valuable in preoperative patient selection, helping to identify those who are more likely to benefit from EVT.

### 4.3 Collateral circulation and distal occlusion condition

Our study also indicated that the condition of the distal end of the occlusion plays a critical role in EVT. Visualization of the distal vessel via retrograde perfusion provides a target for guidewire navigation, facilitating accurate traversal of the occlusion and reducing procedural difficulty. Previous studies have identified poor collateral circulation as a risk factor for hyperperfusion syndrome (34), while robust collateral networks may decrease the incidence of hyperperfusion injury. Additionally, good collateral circulation is associated with higher recanalization success rates (35). Our findings are consistent with previous studies, and these factors play a crucial role in informing our preoperative evaluations.

Based on our statistical findings, we developed a scoring system to identify patients who are most likely to benefit from EVT recanalization. This tool aids in patient selection by quantifying negative predictors, thereby avoiding unnecessary risks in patients with a lower likelihood of procedural success and guiding alternative treatment strategies for them. In clinical practice, clinicians can assign scores to patients based on four readily assessable parameters (occlusion duration, morphology, length, and collateral compensation). Patients with a score ≤ 2 points (e.g., occlusion duration ≤3 months, tapered stump, length ≤ 30 mm, good collaterals) have an over 80% probability of successful recanalization, justifying the treatment. On the other hand, patients with a score > 2 points may require careful consideration. However, since this model was developed using historical data, it may overestimate performance in real-world settings due to selection bias. Additionally, this scoring system does not account for intraoperative factors (e.g., operator experience). Future studies are expected to refine the scoring criteria by integrating high-resolution vessel wall imaging (HR-VWI) and perform external validation in geographically diverse cohorts (e.g., European and North American centers) to assess its generalizability across populations with varying occlusion etiologies and treatment protocols.

This study has several limitations. Due to the retrospective multicenter design and the lack of high-resolution vessel wall imaging (HR-VWI) data, our ability to explore these vessel wall characteristics as potential predictors is limited. Additionally, the retrospective nature of this study may introduce selection bias, and there is a lack of long-term follow-up data. Future prospective studies should expand the sample size, integrate HR-VWI to validate the findings and refine the predictive model, and extend follow-up duration to assess long-term clinical outcomes.

## 5 Conclusion

This study identifies several independent negative predictors of successful recanalization in EVT for NABAO. These predictors include a time interval from diagnosis to surgery greater than 3 months, blunt occlusion stump morphology, an occlusion length exceeding 30 mm, and insufficient or absent distal collateral compensation. Recognizing these factors is critical in preoperative patient selection and may help clinicians optimize treatment strategies by identifying those patients most likely to benefit from endovascular intervention while considering alternative approaches for those with a lower likelihood of success.

## Data Availability

The raw data supporting the conclusions of this article will be made available by the authors, without undue reservation.
